# Dysphagia Lusoria: A Case of an Aberrant Right Subclavian Artery and a Bicarotid Trunk

**DOI:** 10.5402/2011/819295

**Published:** 2011-04-04

**Authors:** A. D. Rogers, M. Nel, E. P. Eloff, N. G. Naidoo

**Affiliations:** Vascular Surgery Unit, Division of General Surgery, Groote Schuur Hospital and University of Cape Town, Cape Town, South Africa

## Abstract

*Dysphagia Lusoria* is dysphagia secondary to an aberrant right subclavian artery that has a retroesophageal course. Adachi and Williams categorized aortic arch anomalies, showing that the right subclavian artery arising in this fashion (as the final branch of the descending aortic arch) is one of the more common. However, this very rarely coexists with a bicarotid trunk. We present such a case as it is manifested in a 36-year-old lady complaining of marked weight loss and dysphagia. The diagnosis remained elusive until a CT scan of the chest was performed; angiography further delineated the pathology. It is believed that the combination of the common carotid origins with the retroesophageal course of the aberrant vessel more frequently accounts for symptoms in the absence of an aneurysm of the origin of the aberrant vessel. Several techniques to manage the aberrant vessel have been described in the literature, but we favoured open ligation and transposition to the right carotid artery.

## 1. Case Report

A 36-year-old women presented to the Gastro-Intestinal Unit of Groote Schuur Hospital with a three-year history of dysphagia. The dysphagia was initially only for solid food and intermittent, but more recently she described a constant “stuck in the throat” foreign body sensation. She also admitted to significant (almost 30 kg) weight loss. She was very anxious and eager to determine the source of the ailment and was concerned about the possibility of malignancy.

Physical examination revealed only evidence of recent weight loss and routine laboratory data were within normal limits.

There were no abnormalities on chest X-ray or ECG. 

**Figure 1:**
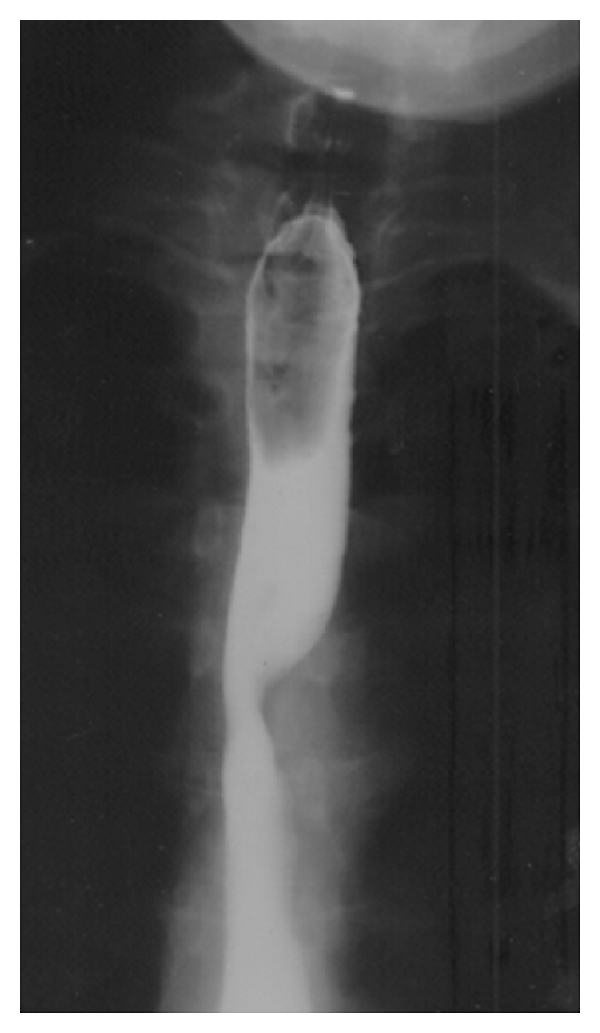
Barium swallow: the characteristic diagonal impression at the level of the third and fourth vertebrae.

Barium swallow demonstrated an indentation at the level of the third thoracic vertebra. A small diverticulum was also reported to be present, just above the aortic arch; see Figure 1. 

No pathology was demonstrated during oesophagoscopy, oesophageal manometry, laryngoscopy, or pharyngoscopy.

A course of Antacids and H2 receptor blockers was initiated for two months, with little benefit. Psychotherapy was considered and antidepressants were even prescribed.

Finally, a CT scan of the chest and abdomen demonstrated an aberrant right subclavian artery, originating from the descending aorta.

**Figure 2:**
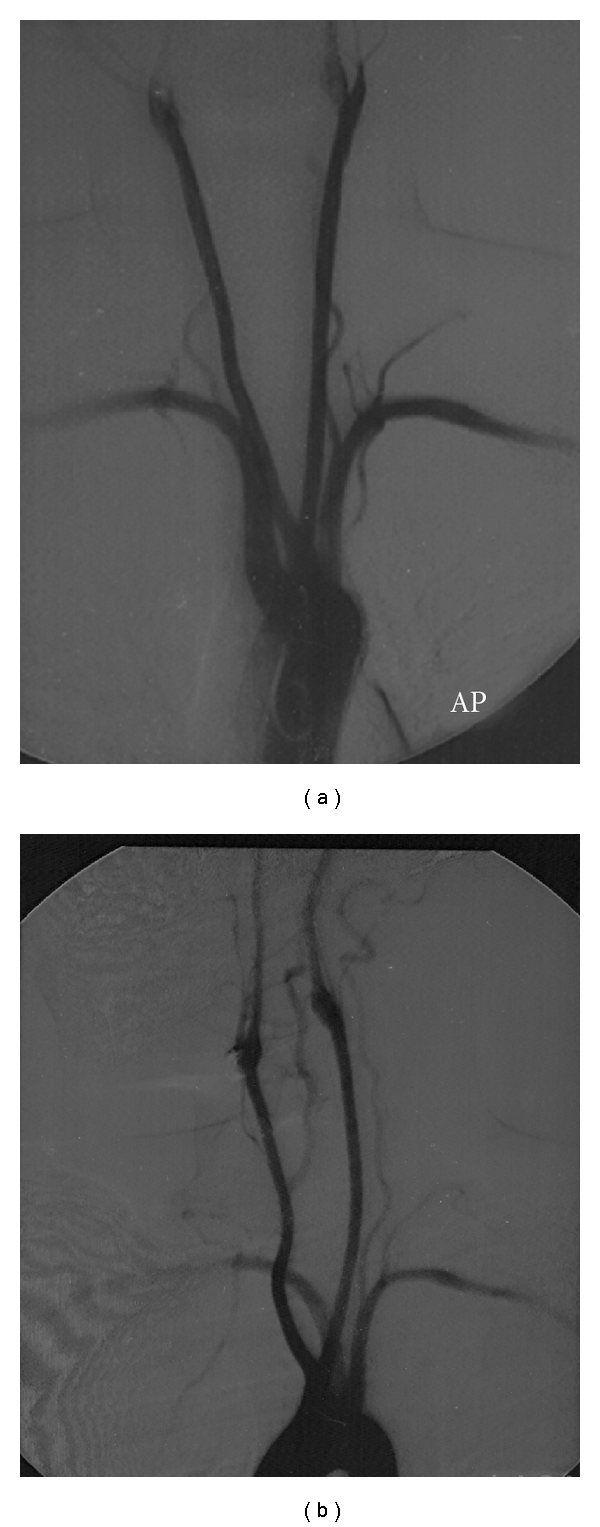
Angiogram: the aberrant right subclavian artery and its course demonstrated on AP and right anterior oblique views. Note also the common origin of the carotid arteries.

**Figure 3:**
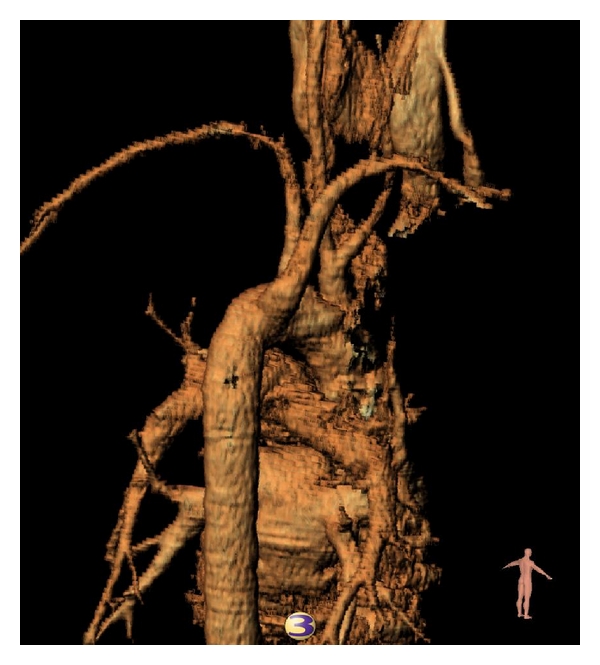
Reconstruction: right posterior oblique view.

The patient was referred to the Vascular Surgery Unit for further investigation and possible surgical intervention. Digital Subtraction Angiogram confirmed the diagnosis. The ARSA originated distal to the origin of the left subclavian artery and coursed through the posterior mediastinum behind the oesophagus. In addition, the angiogram also demonstrated a common origin of the common carotid arteries as a second branch of the arch; see Figures 2 and 3.

**Figure 4:**
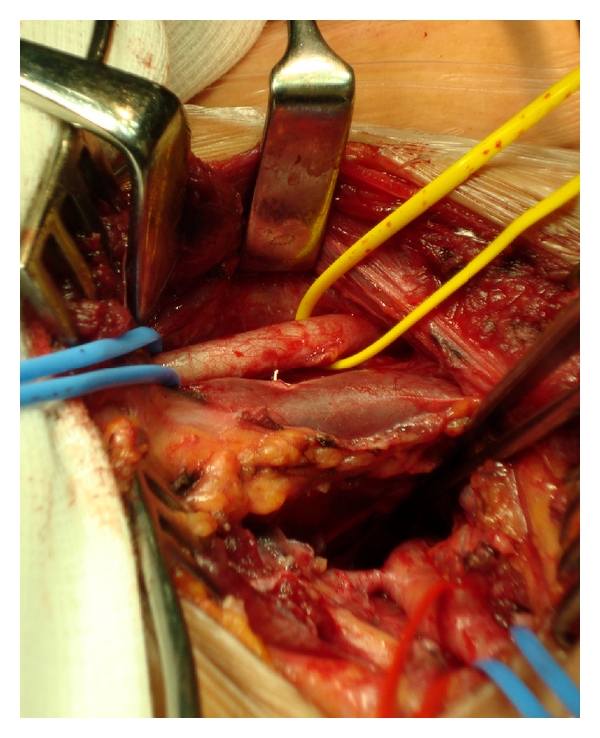
Lateral view via a right supraclavicular incision: note that from above, the right common carotid artery, the right internal jugular vein, and the retroesophageal subclavian artery.

**Figure 5:**
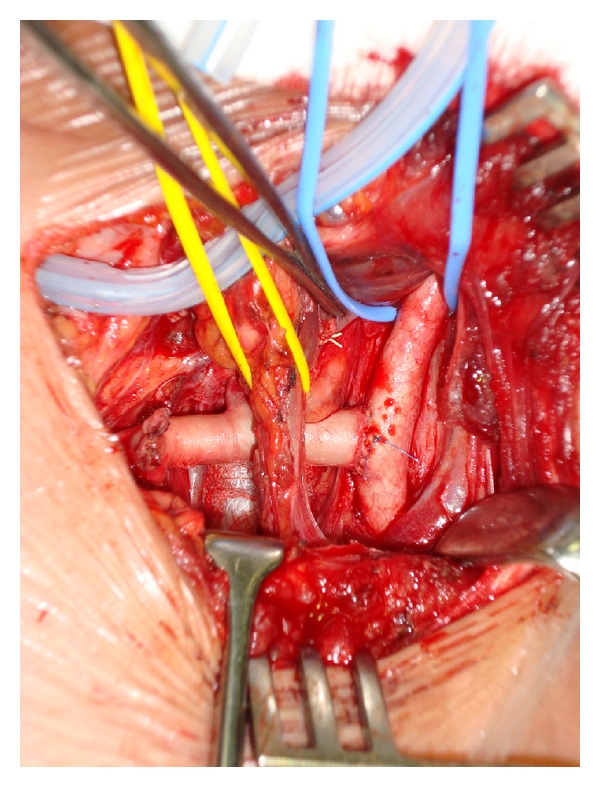
After ligation of the aberrant right subclavian artery and transposition and end-to-side anastomosis to the right common carotid artery.

The patient underwent a right supraclavicular incision and surgical ligation of the ARSA with right subclavian–carotid transposition using an end-to-side anastomosis; see Figures 4 and 5.

The patient had an uneventful postoperative course and remains symptom-free after followup of twelve months.

## 2. Literature Review

The first case of a symptomatic aberrant right subclavian artery (ARSA) was described in the medical literature by Hanuld in 1735 [1].

The term “dysphagia lusoria”, however, was coined by Bayford in 1794 to describe dysphagia secondary to a retroesophageal (aberrant) right subclavian artery (ARSA). He described a lady who died of oesophageal obstruction and resultant emaciation. The condition, in his words “may be called lusoria, from lusus naturae (Latin for “freak of nature”) that gives rise to it” [2]. 

Burckhard Komerell is credited with the first radiological description, in 1936, as the condition had only been diagnosed at postmortem prior to that time. Komerell's name survives as the eponym for the diverticulum sometimes present at the origin of this attenuated vessel. He stated “…the pulsating mass behind the oesophagus does not consist of the right subclavian itself, because the calibre of this vessel is much smaller. Much more likely this mass consists of an aortic diverticulum, from which the right subclavian artery originates” [3].

The incidence of ARSA varies between 0.4 to 1.8% of the population and is probably the commonest significant aortic arch anomaly [4].

### 2.1. The Adachi Williams Classification

In about 80% of individuals, three branches arise from the aortic arch: the brachiocephalic trunk, the left subclavian artery, and the left common carotid artery. This Adachi described as Type A [5].

11% of individuals have an Adachi type B pattern, which consists of a common trunk for the left common carotid and the brachiocephalic artery and therefore has only two aortic arch branches [5].

The next most common type, Adachi C, has a vertebral artery originating proximal to the left subclavian artery as a 4th branch of the arch [5].

The origin of the retroesophageal right subclavian artery as the last branch occurs in between 0.4 and 2% of individuals [4–6].

**Figure 6:**
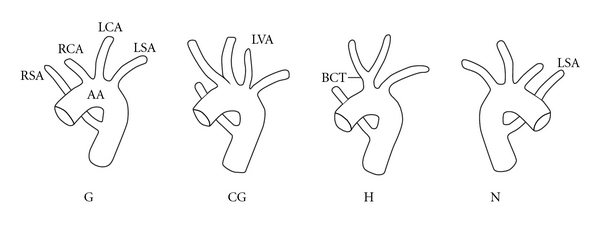
Retroesophageal subclavian anomalies. Note the rare Type H, with the bicarotid trunk.

The Adachi and Williams Classification recognizes four basic morphologies within this group: Types G, CG, H, and N (see Figure 6) [4].

This case report demonstrates features of Adachi H, where the right subclavian artery is anomalous (as in type G), but where the right and left common carotids arise from a common stem or bicarotid trunk.

### 2.2. Embryology

The right subclavian artery develops during the 6th to 8th week of gestation. The proximal part originates from the right 4th aortic arch artery, and the distal part from the right dorsal and right seventh intersegmental arteries [3, 7].

 In these cases, the right 4th aortic arch artery and/or the right dorsal aorta involute cranial to the seventh intersegmental artery—the connection between the aortic sac and the right subclavian artery disappears. The right subclavian artery develops from the right 7th intersegmental artery and the distal segment of the right dorsal aorta. Differential growth shifts the origin cranially and lies close to the origin of the left subclavian artery. It originates dorsally and therefore has a retroesophageal course [3, 7].

The aberrant right subclavian artery stems from the dorsal margin of the aortic arch, between the top of the arch and where it lies against the vertebral column. The proximal part of the artery has a wider diameter than the distal part and the artery passes through the mediastinum in a retroesophageal position. The artery may arise from a diverticulum at the proximal descending aorta, referred to as Kommerell's diverticulum [3, 7].

### 2.3. Presentation

There are several descriptions of childhood dyspnoea resulting from an ARSA, and cases of pneumonia have been ascribed to the condition on the basis of aspiration and dysphagia. Relative tracheal laxity may account for this presentation in childhood [8, 9].

Two thirds of individuals are believed (on the basis of autopsy studies and retrospective studies) to remain completely asymptomatic despite the anomaly. There are a few proposed mechanisms as to why certain individuals become symptomatic:

1. increased oesophageal rigidity with ageing,
2. aneurysm formation,
3. aortic elongation with ageing,
4. the presence of a bicarotid trunk [7, 8].

In a study by Klinkhamer, published in 1966 and reviewing all articles from 1763, it is stated that the aberrant right subclavian artery was found to be associated with a bicarotid truncus (common origin of the right and left carotid arteries) in 85 of 295 cases (29%). In 60% of cases there was a normal origin of the two carotids, and in 10% they were observed to be closer to one another than normal [8]. 

It is believed that the combination of the common carotid origins with the retroesophageal course of the ARSA more frequently accounts for symptoms in the absence of an aneurysm of the origin of the aberrant vessel. In fact, several authors have questioned the relationship between the aberrant vessel (in isolation) and the dysphagia [8]. 

Klinkhamer, for instance, felt that the aberrant artery itself was not an adequate explanation for the dysphagia because some patients have had very large ARSA's, without dysphagia or respiratory symptoms. He maintained that symptoms are usually only present when the left and right carotids arise together or close to one another and therefore prevent the trachea and oesophagus from being bent forward where the ARSA crosses [8].

In 80% of individuals, the Brachiocephalic (BCA) and Left Common Carotid Arteries (LCCA) originate from the arch 4 cm apart. Because the arch lies obliquely and curves from front and the right backward and to the left, the BCA is more ventral than the LCCA origin. If they arise commonly, the two carotids form a “V” that prevents forward or flexion movements of the trachea and oesophagus. These structures are therefore compressed by the SCA posteriorly and by the CC origins anterolaterally [6–10]. 

### 2.4. Investigation [7–11]

Barium swallow may demonstrate the characteristic diagonal impression at the level of the third and fourth thoracic vertebrae.

A pulsating mass may be visualized at endoscopy.

Digital Subtraction Angiogram, CT with contrast, or MRI may confirm the diagnosis and enable one to visualise the arch anatomy.

Motility studies are frequently performed during the diagnostic phase of investigation. A high-pressure zone in the region of the vessel has been described. Manometry cannot be used to diagnose the condition nor has it been of any assistance in distinguishing which patients may benefit from surgery.

### 2.5. Therapy

Several reports have described improvement with conservative therapy. These patients usually had inconclusive findings on manometry. In light of the relative infrequency of symptoms in patients with isolated ARSA, some authors have therefore advocated trials of therapies like prokinetics and antireflux medications.

The majority of symptomatic patients have benefited from surgical intervention. Janssen et al. concluded that in the absence of another cause of the symptoms and after a trial of medical management, surgery should be considered [7]. In 1994, Kieffer et al. reported on 19 patients who underwent surgery, of whom 16 had complete resolution of their symptoms [12]. 

Gross first reported surgical management of this condition in 1946. He described dividing and ligating the ARSA via a left thoracotomy, in a 4 month old infant [13]. Lichter was the first to describe surgery on an adult with this condition in 1963. It is not until the last thirty years that surgery has become the standard therapy for this condition, and several authors have advocated various approaches [14]. Pome et al., in a review of the literature published in 1987, found only twenty reported surgically treated patients [15].

The optimal exposure of the aberrant artery origin is undoubtedly achieved via a left thoracotomy. This is particularly important when the origin of the ARSA is dilated [7, 14].

Simple ligation and division has been noted to be inadequate therapy in a significant number of patients due to the development of subclavian steal syndromes. The onset of this syndrome may be immediately postoperatively or late (described up to seven years later). In addition, cases of gangrene of the right arm have been described following simple ligation of the aberrant vessel [14].

Smith and Pifarré have described reimplanting the right SCA with a graft onto the ascending arch via a left thoracotomy. This is a technically challenging exercise and involves passing the Right SCA and the graft from the posterior mediastinum to the anterior, as well as deep anastomoses [16, 17].

The anastomosis of the RSCA to the ascending arch is much easier to perform via a right posterolateral thoracotomy, as described by authors such as Bailey et al. [18].

Pifarré et al. identified a problem with the right thoracotomy approach in one case report. If the artery is not divided close to its origin, thrombosis of the stump may lead to the persistence of dysphagia [17].

Schumacker described performing an end-to-side anastomosis of the R SCA with the right carotid artery via a median sternotomy. This has been the method described by at least three further surgeons. However, this anterior approach also provides suboptimal control during the dissection and division of the vessel. The possibility of transient cerebral ischaemia and the consequences of damage to an atherosclerotic carotid artery exist with this approach. Mok et al. therefore recommended that the divided aberrant right SCA should be anastomosed to the aortic arch, with or without an interposition graft [10].

Orvald and Kunlin advocated a cervical approach, but Kunlin described significant haemorrhage in a patient when attempting to perform the procedure via a cervical incision and had to resort to a median sternotomy. We have already highlighted the potential complication of residual dysphagia in cases where a long stump is left [15].

Syders reported a combined approach using both cervical and left carotid approaches to reimplant the right SCA onto the right carotid. This, while considerably safer, involves having to reposition the patient for the second incision [15].

Lemire et al. described a transternal approach for the division and reimplanting of the Right SCA to the ascending aorta. The incidence of pain and pulmonary complications are probably higher, but this approach has cosmetic advantages [19].

Pome et al. recommended that a right thoractomy may be employed for patients without significant ectasia of the origin of the Right SCA; patients with ectasia should undergo a left thoracotomy and cervical incision [15].

The importance of dividing the stump proximally is again highlighted in the paper by Pome. He advocated using the aorta rather than the carotid to avoid a possible subclavian steal syndrome [15]. 

Janssen et al. reports six cases of dysphagia lusoria diagnosed and managed between 1992 and 1997. Three patients responded to either medical management (antacids, etc.), or dietary modification. One patient underwent a right carotid-subclavian end to side bypass via a right supraclavicular approach. A persistent RSCA stump may account for his occasional residual dysphagia for solid food. Two patients underwent a two incision approach (i.e., thoracotomy and cervical incision) [7].

 Kieffer et al. have the largest single-centre series of patients who have received therapy for symptomatic or aneurismal aberrant subclavian arteries. They divided their patients into four distinct subgroups:

1. dysphagia lusoria without aneurysm,
2. symptomatic occlusive disease of the artery,
3. aneurysmal disease of the artery itself,
4. aneurysmal disease of the thoracic aorta or origin of the aberrant artery.

It would appear sensible to manage all patients with aneurismal disease with stent grafting in light of the high rupture rate (22.6%) and consequent mortality rate (100%) independent of the diameter of the aneurysm. The perioperative rate of patients undergoing surgical repair of these aneurysms was 26.9% [12].

There are few reports in the literature of endovascular or hybrid approaches to this pathology. Although long-term results are still pending, initial results are promising. Shennib and Diethrich have described minimally invasive, hybrid endovascular approaches. A right supraclavicular approach was used to perform a right carotid-subclavian bypass prior to division of the right SCA. They then deployed an occluder in an antegrade fashion into the proximal end of the right SCA in order to maintain good control of the deployment and to avoid embolization of the occluder into the aortic arch from a retrograde approach. A right femoral artery access was used via a 9F sheath [20].

Endoluminal grafts have also been used with some success in the presence of aneurysm of the ARSA origin. In these cases it may be necessary to consider performing a further anastomosis between the left carotid and subclavian artery if overstenting was intended.

Kopp et al. have reported a series of six patients managed by a variety of techniques during a seventeen year period. One of the patients had a covered wall stent with an occluded proximal stent graft lumen inserted via a transbrachial approach. The right SCA was then anastomosed to the right common carotid artery [21].

## 3. Conclusions

We present a case where the right subclavian artery originated as the final branch of the aortic arch, coursed posterior to the oesophagus, and coexisted with a bicarotid trunk.

It is believed that the combination of the common carotid origins with the retroesophageal course of the aberrant vessel more frequently accounts for symptoms in the absence of an aneurysm of the origin of the aberrant vessel.

Several techniques to manage the aberrant vessel have been described in the literature with varied success.

Strategies:

1. conservative,
2. medical,
3. surgical.

Surgical Access:

1. left or right thoracotomy,
2. median sternotomy or trans-sternal,
3. cervical,
4. right supraclavicular,
5. endovascular,
6. hybrid.

Reconstructions:

1. simple ligation and division,
2. right subclavian artery to ascending arch,
3. right subclavian artery to carotid artery,
4. stent-grafting for aneurismal disease.

We favoured open ligation and transposition to the right carotid artery via a right supraclavicular approach. We selected this approach as it has the lowest associated morbidity, but acknowledge the inherent risk of recurrent symptoms should the aberrant vessel not be ligated close enough to its origin.
